# A Defect in Medical Education

**Published:** 1897-10-09

**Authors:** 


### The Hospital

A Journal of

The Medical Sciences and Hospital Administration.

Vol. XXIII.—No. 576.] [Oct. 9, 1897.

### A Defect in Medical Education.

It would be difficult to frame a more serious indictment of the whole system of medical education which is at present in vogue in England than is contained in the introductory address delivered at St. George's Hospital last week by Dr. Patrick Manson.

He points out what, indeed, is evident enough to all who have to do with the students of the day, that the curriculum is decided by the examinations, and that what a student is not examined in, that he will not learn. And small blame to him. The whole plan of the medical student's life is mapped out for him ; he must go through the mill, and if he would emerge safely, he must not step a hair's breadth out of the prescribed course. The responsibility, then, for the product turned out by this educational mill lies entirely upon those who made it, and if practitioners emerge absolutely ignorant of whole groups of diseases, great as is the wrong done to them, and great as is the danger to which the public are thereby exposed, the blame should not fall on the poor students, but upon those who are responsible for the medical curriculum of the day.

More than a fifth of all the medical practitioners turned out by the schools of Great Britain and Ireland either practise in hot climates, or, being in the army or navy, may be called upon at any time to do so, yet Dr. Patrick Manson declares that the course of instruction usually received in these very schools is utterly inadequate to qualify for tropical practice.

After giving many striking examples of the trials and difficulties with which a man with an English medical education may, nay will, find himself involved when he attempts to practise his profession in the tropics, he goes on to point out that in this matter he is not speaking of the past but of the condition of affairs current at the present day. Beri-beri, says Dr. Manson, although a tropical disease, is very common in the port of London, especially among the Lascars and seedy boys who form the bulk of the crews of the large steamers trading to the East. Cases of this disease are often brought to the Seamen's Hospital. They come in with written medical recommendations, being sent as cases of heart disease, kidney disease, "almost anything but beri-beri." Yet the ship-surgeons who make out these certificates are usually men fresh from the schools and from their examinations, and presumably well up in all that is newest in medicine. "Here," he says, "is a verbatim enumeration of some of their diagnoses : anasarca, rheumatism, pericarditis and fits, dyspnœa, cardiac disease, debility, asthma and anasarca, Bright's disease, locomotor ataxy, myelitis, tachycardia, apoplexy, paraplegia, progressive muscular atrophy, and, of course, hysteria. I transcribe these diagnoses from the admission registers. Not once in a dozen instances is the diagnosis correct." It is impossible to pass over such a charge as this, brought so formally against those responsible for medical teaching in London and other centres of medical education. It would seem, in fact, that while students are being taught many smatterings of many sciences, they are not being properly instructed in the practical details of their purely medical work, and are being turned loose as qualified medical practitioners, although quite ignorant of many of the diseases the treatment of which may form a large part of their every-day duty.

Now, the reason why such a state of affairs exists is stated to be that the leaders of the profession—those who man our hospitals, who fill the teaching chairs, who examine for degrees, who grant licences to practise, who make the regulations for the education of the youth of the profession—are, in almost every instance, men who themselves have never felt the necessity for a wider and more practical knowledge of the diseases peculiar to warm climates.

This is equivalent to saying that our leaders not only confine their teaching to the little circle of knowledge that comes personally before them, but that they so arrange the curriculum as to block the way to the embryo medicos of the future extending their search into wider fields. The charge is a serious one, especially when we bear in mind that practitioners go out into the world ticketed as "qualified," and entered on a Government register, which is maintained at great expense (out of the pockets of the profession be it said), for this sole reason, as expressed in the preamble of the Medical Act, that "it is expedient that persons requiring medical aid should be enabled to distinguish qualified from unqualified practitioners." The entry of a man's name on the register is to all intents and purposes a Government guarantee that he has been so educated as to be qualified for practice; and yet, notwithstanding all the cries we hear about Imperialism, and the undoubted facts that our fellow subjects occupy an immense belt of tropical country, extending almost round the globe, and that one in five of all our "qualified" men have to practise in these hot regions, we find that the machinery for giving this highly honourable distinction is so badly constructed and so carelessly managed, that men are turned out as "qualified," although their knowledge extends only to such diseases as occur in one corner of our great empire.

